# Cryoablation of renal tumors: long-term follow-up from a multicenter experience

**DOI:** 10.1007/s00261-021-03082-z

**Published:** 2021-04-29

**Authors:** Fulvio Stacul, Camilla Sachs, Fabiola Giudici, Michele Bertolotto, Michele Rizzo, Nicola Pavan, Luca Balestreri, Oliviero Lenardon, Alessandro Pinzani, Lisa Pola, Calogero Cicero, Antonio Celia, Maria Assunta Cova

**Affiliations:** 1 Department of Radiology, Ospedale Maggiore, Trieste, Italy; 2 Department of Radiology, University of Trieste, Trieste, Italy; 3 Unit of Biostatistics, Epidemiology and Public Health, Department of Cardiac, Thoracic, Vascular Sciences and Public Health, University of Padova, Padova, Italy; 4 Unit of Biostatistic, Department of Medical, Surgical and Health Sciences, University of Trieste, Trieste, Italy; 5 Department of Urology, University of Trieste, Trieste, Italy; 6 SOC Radiologia, Centro di Riferimento Oncologico Aviano, Aviano, Italy; 7 SOC Urologia, Ospedale Santa Maria degli Angeli, Pordenone, Italy; 8 UO di Radiologia Dolo, AULSS 3, Serenissima, Italy; 9 UO Urologia Dolo, AULSS 3, Serenissima, Italy; 10 Department of Radiology, San Bassano Hospital, Bassano del Grappa, Italy; 11 Department of Urology, San Bassiano Hospital, Bassano del Grappa, Italy

**Keywords:** Kidney neoplasm, Cryoablation, Ablation techniques, Multicenter study

## Abstract

**Purpose:**

To retrospectively investigate long-term outcomes of renal cryoablation from a multicenter database.

**Methods:**

338 patients with 363 renal tumors underwent cryoablation in 4 centers in North-Eastern Italy. 340/363 tumors (93.7%) were percutaneously treated with CT guidance. 234 (68.8%) were treated after conscious sedation, 76 (22.3%) under local lidocaine anesthesia only and 30 (8.8%) under general anesthesia. Treatment efficacy and complication rate considered all procedures. Oncologic outcomes considered a subset of 159 patients with 159 biopsy proven renal cell carcinoma.

**Results:**

Mean tumor size was 2.53 cm. Technical success was achieved in 355/363 (97.8%) treatments. Treatment efficacy after the first treatment was achieved in 348/363 (95.9%) tumors. Statistical analysis revealed a significant lower treatment efficacy for ASA score >3, Padua score >8, tumor size >2.5 cm, use of >2 cryoprobes, presence of one single kidney. In the subset of 159 patients, recurrence-free survival rates were 90.5% (95% CI 83.0%, 94.9%) at 3 years and 82.4% (95% CI 72.0%, 89.4%) at 5 years; overall survival rates were 96.0% (95% CI 90.6%, 98.3%) at 3 years and 91.0% (95% CI 81.7%, 95.7%) at 5 years; no patient in this subset developed metastatic disease. Clavien-Dindo >1 complications were recorded in 14/369 procedures (3.8%) and were related to age >70 years, tumor size >4 cm and use of >2 cryoprobes.

**Conclusion:**

Cryoablation performed across four different centers in a large cohort of predominantly small renal tumors showed that this technique provides good recurrence-free survival rates and overall survival rates at three- and five-year with very low major complications rate.

**Electronic supplementary material:**

The online version of this article (10.1007/s00261-021-03082-z) contains supplementary material, which is available to authorized users.

## Introduction

The use of thermal ablation for treatment of T1 renal cell carcinoma (RCC) has been increasing over the last years, but there are not enough data supporting its widespread use and physicians should be aware of the lack of high-quality evidence in this setting [1, 2]. As a result, current guidelines by Urological and Oncological Societies are very cautious in recommending thermal ablation [3–7].

Long-term efficacy results of RCC thermal ablation start being collected and 5-year outcomes of large series treated with cryoablation in single center teaching hospitals, often under general anesthesia, are available [8–10]. Cryoablation with local anesthesia and conscious sedation was reported as an alternative modality [11–13] and shortened perioperative time and hospital stay [12]. To our knowledge cryoablation under local anesthesia only was not reported so far.

In this paper, we considered cryoablations performed in both teaching and non-teaching general hospitals using the same technique and performing the treatment without general anesthesia, with the aim of retrospectively investigating long-term outcomes of renal cryoablation from a multicenter database.

## Materials and methods

### Patients

This multicenter retrospective study involved 4 centers in North-Eastern Italy (center 1: University Hospital of Cattinara – Trieste; center 2: Oncologic Reference Center of Aviano; center 3: General Hospital of Dolo; center 4: General Hospital of Bassano). These centers cooperate within a research group involving centers performing cryoablation in North-Eastern Italy (Cryoablation Focus Group). Data collection considered 12 years of activity (Nov 2007-Sep 2019) for a total of 338 patients with 363 renal tumors. All patients gave informed consent for cryoablation and approval by the institutional review board was obtained.

The large majority of cases were discussed in Multidisciplinary Meetings involving Urologists, Radiologists, Oncologists and Radiotherapists. Cryoablation was primarily offered to patients with contraindications to surgery or with significant comorbidities. Such patients represented the large majority of cases. Some patients underwent cryoablation because of a single functioning kidney or because of presence of more than one small renal tumor. Few patients underwent cryoablation because of patient preference. These indications for ablation were in agreement with CIRSE Guidelines [14]. Uncorrectable coagulopathy was considered an absolute contraindication. Anatomical conditions that would hinder percutaneous access to the lesion were considered relative contraindications.

Following the procedure patients usually spent one night in the Urology Department, unless unexpected complications occurred.

### Procedures

All cryoablations were performed with the Galil Medical/BTG cryoablation system. Treatments distribution was as follows: 142 in center 1, 83 in center 2, 71 in center 3 and 67 in center 4. 17G cryoprobes were placed within the tumor aiming at a radical treatment in one single session with an adequate safety margin (at least 5 mm). Type and number of cryoprobes varied according to tumor size and location. Cryoablations were all performed with the same technique, with a double freeze-thaw cycle. Saline hydrodissection for mechanical displacement of the bowel was regularly used when the distance between the tumor and the bowel was 1 cm or less.

340/363 (93.7%) tumors were percutaneously treated with computerized tomography (CT) guidance. The type of sedation was based on the preference and experience of anesthesiologists in the different centers. 30 (8.8%) of the percutaneously treated tumors were treated under general anesthesia. These 30 tumors represent the first cases treated with cryoablation in center 3. Later on anesthesiologists from this center preferred conscious sedation. 234 (68.8%) tumors were treated after conscious sedation and 76 (22.3%) tumors from centers 1 and 4 under local lidocaine anesthesia only, as for the past few years the anesthesiologists have considered unfit for local anesthesia only uncooperative patients or patients potentially unable to remain prone throughout the cryoablation procedure. Center 4 additionally treated 23 (6.3%) tumors with laparoscopic guidance in the operating theater under general anesthesia. The large majority of these tumors represent the first cases treated with cryoablation in center 4. Few patients underwent cryoablation through a laparoscopic approach later when a percutaneous access to the lesion was considered extremely difficult.

Percutaneous biopsy of the lesion was performed before cryoablation or at the same time of the procedure in 326 (89.8%) cases. In the majority of cases (248/326-76%) biopsy was performed just before cryoablation, before placement of ablation probes. The same track was often used for biopsy needle and one cryoablation probe.

### Follow-up

The follow-up in the four centers considered contrast enhanced magnetic resonance (CE-MRI) or contrast enhanced CT (CE-CT) 6 months, 1 year, 2 years, 3 years, 4 years and 5 years after ablation. CE-MRI was usually preferred over CE-CT. Centers 1 and 4 additionally performed contrast enhanced ultrasound (CEUS) 1 month after cryoablation, while the other 2 centers performed CE-MRI or CE-CT 1 month after the procedure. This different approach is explained by the large experience with CEUS of centers 1 and 4. In these centers patients also had pre-procedure CEUS for comparison with one-month follow-up imaging [15].

Technical success was defined as a complete treatment of the lesion according to the protocol. Treatment efficacy was defined as a successful ablation without residual tumor by one month. Residual tumor was defined as persistent evidence of enhancement within the ablated lesion on the one-month follow-up imaging. Tumor recurrence was defined as a new enhancement of the ablated lesion during the follow-up following a previously documented successful treatment. All such definitions followed the Cardiovascular and Interventional Radiological Society of Europe (CIRSE) Guidelines [14]. Most patients with residual disease or local recurrence underwent another cryoablation.

Radiology reports of follow-up exams served as a reference standard for technical success, treatment efficacy, presence of residual tumor or tumor recurrence.

Complications were classified according to the Clavien-Dindo system [16]. Grade 3 or greater are considered major complications (grade 3: requiring surgical, radiologic or endoscopic intervention; grade 4: life-threatening condition requiring intensive-care unit management; grade 5: death). Complications were recorded retrospectively, after check of medical records.

### Database

All 4 centers retrospectively checked data in relation with the procedures and introduced them into a database using the RedCap sw. The following 17 variables were considered: n. of the center, gender, age, body mass index (BMI), ASA (American Society of Anesthesiology) score, single kidney, tumor size, PADUA score [17], tumor histology, history of previous RCC and its treatment, n. of tumors treated in one session, approach (percutaneous or laparoscopic), type of anesthesia, number and characteristics of cryoprobes, complications, serum creatinine (at baseline and 24-48 h post-procedure), imaging follow-up recording complete response/persistence/recurrence.

### Statistical analysis

Categorical variables were reported as percentages, continuous variables were summarized as mean ± standard deviation or median and range as appropriate, according with the data distribution. Normality of the continuous variables was tested using the Shapiro–Wilk test.

We analyzed factors that could be related to treatment efficacy and complication rates of procedures: variables of interest were compared using the Chi-squared or Fischer Exact Test for categorical parameters, and with the Mann-Whitney test or t-student test for continuous ones.

Recurrence–free survival (RFS) was defined as the interval between the date of the first cryoablation and the date of the first local recurrence, with censoring at the last examination date for patients who did not have a local recurrence. Metastasis free survival (MFS) was defined as the percentage of patients without metastatic lesions from the ablated tumor. Overall survival (OS) was defined as the time between the date of the first cryoablation and the date of death, or the date of the last follow-up for alive patients. RFS, MFS and OS rates were estimated using the Kaplan-Meier method. The median follow-up was computed for censored patients, excluding patients with the events of interest (reverse Kaplan-Meier method)

RFS was estimated using the Kaplan–Meier approach. The association between variables and survival distribution was tested using univariate Cox proportional hazard models (after verification of proportional hazard assumptions).

Statistical analysis for treatment efficacy and complication rates considered all ablative procedures. Statistical analysis for RFS, MFS, and OS considered patients with biopsy proven RCC and without heritable renal cancer syndromes / without history of previous RCC.

All statistical analyses were performed with R software (version 3.5.0) and p-value <0.05 was considered to indicate a statistically significant difference.

## Results

338 patients (242 males (71.6%), 96 females (28.4%), mean age 73 years, range 39-90 years) with 363 renal tumors underwent cryoablation in the four centers. Demographic data including BMI and ASA score are reported in Table 1. Additionally, 36 patients had a single kidney and one patient underwent treatment of a tumor in a transplanted kidney.

**Table 1:** Clinical features for 338 patients with renal masses and for the subset of 159 patients with biopsy proven renal cell carcinoma

	All patients (*n* = 338)	Patients with biopsy proven RCC (*n* = 159)
*Age*		
Mean (SD)	73 (9)	73 (9)
Median (Min–Max)	75 (39–90)	75 (43–89)
*Gender*		
Male (*n*, %)	242 (71.6%)	114 (71.7%)
Female (*n*, %)	96 (28.4%)	45 (28.3%)
*BMI*		
Mean (SD)	24.5 (3.6)	25.9 (3.0)
Median (Min–Max)	26.2 (18.6–40.1)	25.7 (18.6–36)
*ASA*		
Mean (SD)	2.6 (0.6)	2.6 (0.6)
Median (Min–Max)	3 (1–4)	3 (1–4)
*ASA*		
ASA score 1 (*n*, %)	13 (3.8%)	8 (5.0%)
ASA score 2 (*n*, %)	127 (37.6%)	59 (37.1%)
ASA score 3 (*n*, %)	184 (54.4%)	88 (55.4%)
ASA score 4 (*n*, %)	14 (4.1%)	4 (2.5%)
*Single kidney* (*n*, %)	36 (10.7%)	0 (0.0%)
*Transplanted kidney *(*n*, %)	1 (0.30%)	1 (0.63%)
*History of RCC*		
No (*n*, %)	284 (84.0%)	//
Yes (*n*, %)	40 (11.8%)	//
Hereditary syndrome (*n*, %)	14 (4.1%)	//

*RCC* Renal Cell Carcinoma, *SD* Standard Deviation, *BMI* Body Mass Index, *ASA* American Society of Anesthesiology

10 patients had more than one tumor at the onset and 6 patients developed other tumors following the first treatment, therefore they underwent more than one treatment. Additionally, 15 lesions were retreated, because of tumor persistence after the first treatment or because of recurrence.

The mean diameter of the 363 treated lesions was 2.53 cm (range 0.6-5.3 cm). 336 (92.6%) of them were 4.0 cm or smaller (T1a tumors) and 27 (7.4%) were >4.0 cm (T1b tumors). Tumors characteristics including PADUA score are reported in Table 2. A mean of 2.48 cryoprobes was used (range 1-8).

**Table 2:** Tumor characteristics for 338 patients with renal masses and for the subset of 159 patients with biopsy proven renal cell carcinoma

	All patients (*n* *=* 338)	Patients with biopsy proven RCC (*n* = 159)
*Tumor size* (mm)		
Mean (SD)	25.3 (9.6)	26.1 (9.6)
Median (Min–Max)	25 (6–53)	25 (8–53)
*Location*		
Anterior (*n*, %)	91 (26.9%)	49 (30.8%)
Posterior (*n*, %)	247 (73.1%)	110 (69.2%)
*Location*		
Endophytic (*n*, %)	63 (18.6%)	33 (20.8%)
Esophytic (*n*, %)	160 (47.3%)	72 (45.3%)
Partially esophytic (*n*, %)	115 (34.0%)	54 (33.9%)
*Padua score*		
Mean (SD)	7.8 (1.4)	7.9 (1.4)
Median (Min–Max)	8 (6–12)	8 (6–12)
*Padua score*		
6–7 (*n*, %)	156 (46.4%)	67 (42.4%)
8–9 (*n*, %)	136 (40.5%)	71 (44.9%)
> = 10 (*n*, %)	44 (13.1%)	20 (12.7%)
*N° of tumors*		
1 (*n*, %)	329 (97.3%)	159 (100.0%)
> 1 (*n*, %)	9 (2.7%)	0 (0.0%)
*Histotype*		
Clear cells (*n*, %)	//	103 (64.8%)
Papillary (*n*, %)	//	35 (22.0%)
Chromophobe (*n*, %)	//	12 (7.5%)
Unspecified adenocarcinoma (*n*, %)	//	9 (5.7%)

*RCC* Renal Cell Carcinoma, *SD* Standard Deviation

**Fig. 1:**
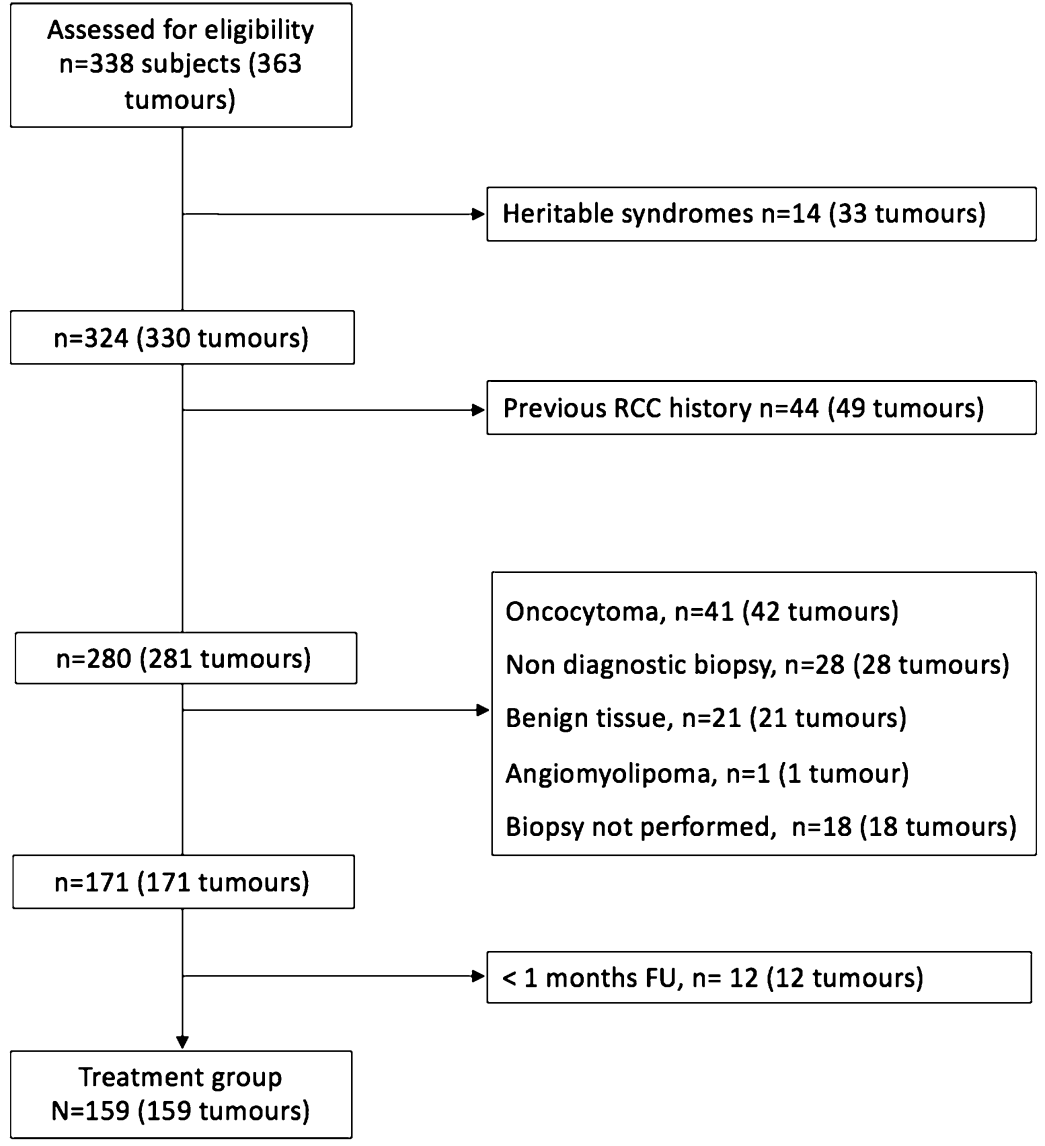
Patient selection flowchart. (*RCC*: Renal Cell Carcinoma; *FU*: Follow-Up)

The subset analysis of biopsy proven RCCs excluded patients with heritable renal cancer (*n* = 12 (27 tumors)), patients with previous surgical treatment of RCC (*n* = 72 (77 tumors)), patients with inadequate or benign histologic findings (*n* = 70 (75 tumors)), patients with lesions that did not undergo biopsy (n = 13 (13 tumors)) and patients who were lost at the follow-up (*n* = 12 (12 tumors)) (Fig. 1). We recorded 159 biopsy proven RCCs in 159 patients following this analysis, including 147 T1a tumors and 12 T1b tumors. Demographic data and tumor characteristics in this group are listed in Table 1 and in Table 2, respectively.

Technical success was achieved in 355/363 (97.8%) treatments. In the remaining 8 procedures different features prevented a successful result. In 3 procedures one cryoprobe had a technical failure in spite of the pre-procedural check. In 2 cases the patient treated with local anesthesia only could no longer tolerate the treatment after a long procedure with treatment of multiple tumors. In one patient with multiple tumors, a very long procedure was stopped and treatment of one residual lesion was postponed. In one case the close proximity of the tumor to the renal pelvis prevented a full treatment. Finally, in one patient treatment had to be stopped due to the onset of bradycardia. 6 out of these 8 lesions were successfully retreated with cryoablation, one patient entered an active surveillance protocol after multidisciplinary decision and the last one was lost at the follow-up.

**Fig. 2:**
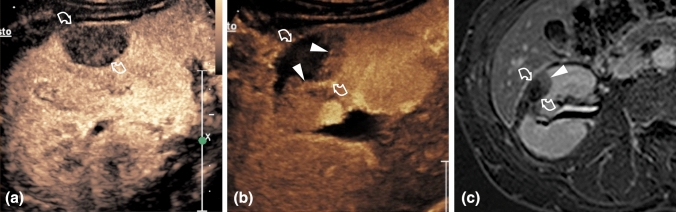
Incomplete tumor cryoablation. **a** Before cryoablation CEUS shows a homogeneously vascularized renal tumor (curved arrows). **b** A crescent enhancing area consistent with residual tumor (arrowheads) is present one month after the treatment of the lesion (curved arrows). **c** Gadolinium-enhanced subtracted image six months after the treatment shows enhancing tumor tissue (arrowhead) and confirms incomplete ablation of the lesion (curved arrows)

Treatment efficacy after the first treatment was achieved in 348/363 (95.9%) tumors. Besides the 8 technical failures, 7 residual lesions were detected at the one-month follow-up (Fig. 2). Repeated cryoablation was performed in 4 out of these 7 cases and was successful in 2 of them, while the other 2 patients who experienced residual lesion after two cryoablations finally underwent surgery. 2 of the remaining 3 patients underwent active surveillance after multidisciplinary decision, while the last one was lost at the follow-up. Treatment efficacy did not significantly differ between the 4 centers (p = 0.48, Chi-squared test). Finally, treatment efficacy after the second treatment was achieved in 356/363 (98.1%) tumors. Statistical analysis revealed a significant lower treatment efficacy for ASA score > = 4, Padua score > = 9, tumor size > = 2.5 cm, use of >2 cryoprobes, presence of one single kidney/transplanted kidney (Table 3).

**Table 3:** Analysis of variables predicting treatment efficacy of 363 renal tumors

Variables	Treatment efficacy after primary ablation (*n* = 348)	No treatment efficacy after primary ablation (n = 15)	*p*-value
*Center*			0.48
n. 1 (*n*, %)	135 (95.1%)	7 (4.9%)	
n. 2 (*n*, %)	66 (98.5%)	1 (1.5%)	
n. 3 (*n*, %)	75 (93.8%)	5 (6.3%)	
n. 4 (*n*, %)	69 (97.2%)	2 (2.8%)	
*Gender*			0.28
Male (*n*, %)	253 (96.6%)	9 (3.4%)	
Female (*n*, %)	95 (94.1%)	6 (5.9%)	
*Age*			0.73
Median (Min–Max)	74.6 (39.1–89.4)	75.6 (45.6–89.6)	
*BMI*			0.63
Median (Min–Max)	26.2 (18.6–40.1)	27.1 (20.5–37.4)	
*ASA score*			0.02*
<4 (*n*, %)	336 (96.6%)	12 (3.4%)	
> = 4 (*n*, %)	12 (80.0%)	3 (20.0%)	
*Single kidney or transplanted kidney*			0.02*
Yes (*n*, %)	43 (89.6 %)	5 (10.4%)	
No (*n*, %)	305 (96.8%)	10 (3.2%)	
*History of RCC*			0.71
No (*n*, %)	274 (96.1 %)	11 (3.0%)	
Yes (*n*, %)	43 (95.6%)	2 (4.4%)	
Hereditary syndrome (*n*, %)	31 (94.0 %)	2 (6.0%)	
*Tumor size* (mm)			0.002*
*Median (Min–Max)*	24 (6–53)	34 (15–50)	
<25 mm (*n*, %)	176 (98.9%)	2 (1.1%)	
> = 25 mm (*n*, %)	172 (93.0%)	13 (7.0%)	
*Location*			0.21
Endophytic (*n*, %)	73 (96.1%)	3 (3.9%)	
Esophytic (*n*, %)	162 (97.6%)	4 (2.4%)	
Partially esophytic (*n*, %)	113 (93.4%)	8 (6.6%)	
*Padua score*			0.01*
*Median (Min–Max)*	8 (6–12)	9 (7–11)	
<9 (*n*, %)	247 (97.6%)	6 (2.4%)	
> = 9 (*n*, %)	101 (91.8%)	9 (8.2%)	
*Number of tumors for treatment session*			0.25
1 (*n*, %)	327 (96.2%)	13 (3.8%)	
>1 (*n*, %)	21 (91.3%)	2 (8.7%)	
*Anesthesia*			0.93
Local (*n*, %)	74 (97.4%)	2 (2.6%)	
Sedation (*n*, %)	223 (95.3%)	11 (4.7%)	
General–Laparo (*n*, %)	22 (95.7%)	1 (4.3%)	
General–Percutaneous (*n*, %)	29 (96.7%)	1 (3.3%)	
*Approach*			0.96
Percutaneous (*n*, %)	326 (95.9%)	14 (4.1%)	
Laparoscopic (*n*, %)	22 (95.7%)	1 (4.3 %)	
*Number of cryoprobes*			0.02*
*Median (Min–Max)*	2 (1–8)	3 (2–7)	
< = 2 (*n*, %)	217 (97.8%)	5 (2.2%)	
>2 (*n*, %)	131 (92.9%)	10 (7.1%)	
*Baseline Serum Creatinine* (mg/dl)			0.10
Median (Min–Max)	1.01 (0.50–6.2)	1.01 (0.51–7.0)	
Baseline Serum Creatinine**			0.18
<1.30 mg/dl (*n*, %)	265 (96.7%)	9 (3.3%)	
> = 1.30 mg/dl (*n*, %)	69 (93.2%)	5 (6.8%)	

*RCC* Renal Cell Carcinoma, *BMI* Body Mass Index, *ASA* American Society of Anesthesiology

**15 missing data

**Fig. 3:**
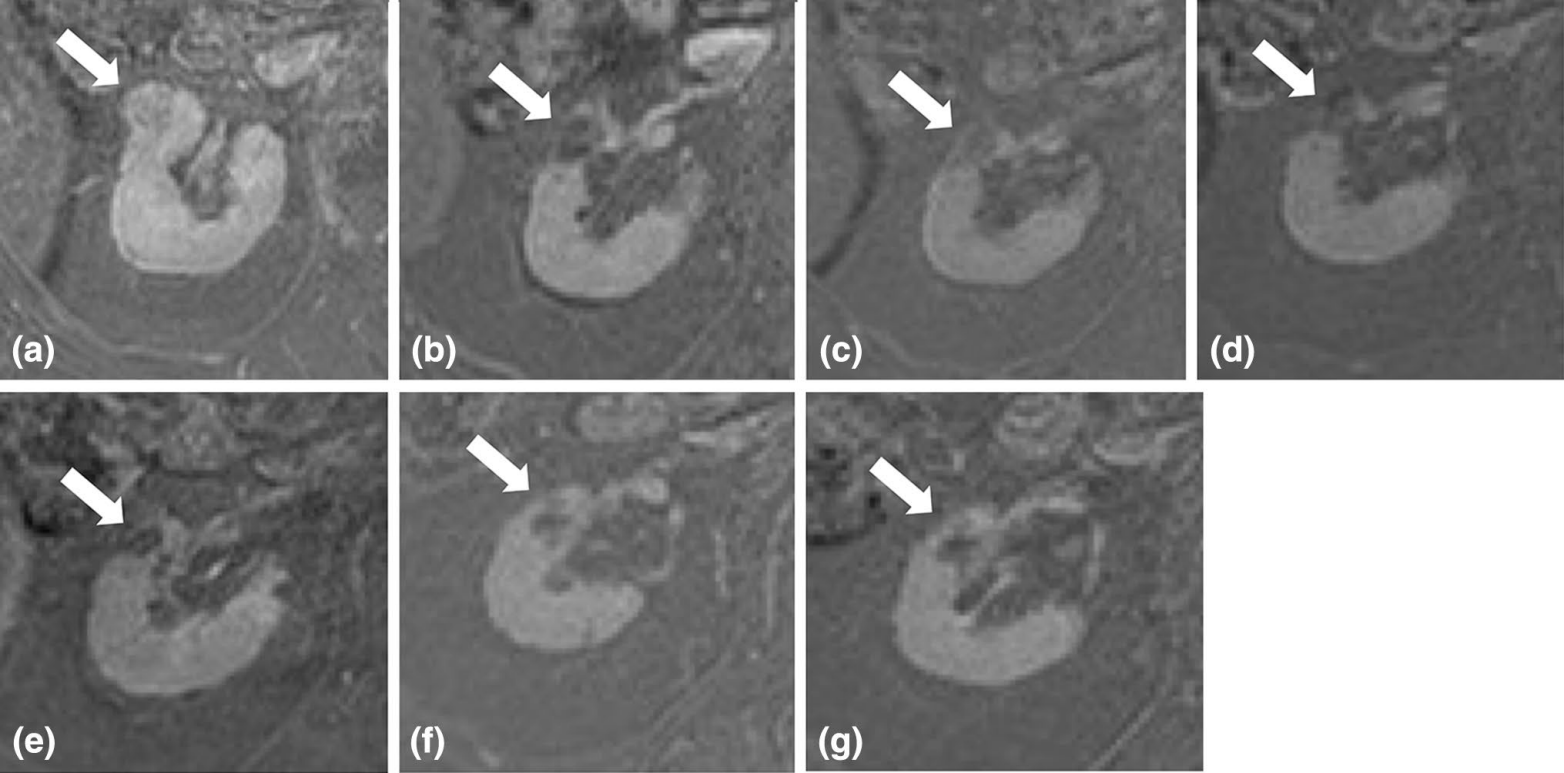
Normal CE MR imaging appearance following cryoablation of a RCC. **a** Pre-ablation fat-suppressed T1-weighted CE MRI shows **a** 2.5 cm enhancing mass in the mid portion of the right kidney, located anteriorly (arrow). **b**–**g** Follow-up CE MRI 6 months, 1 year, 2 years, 3 years, 4 years and 5 years after cryoablation show lack of enhancement within the ablation zone together with progressive shrinkage of this area (arrows)

**Fig. 4:**
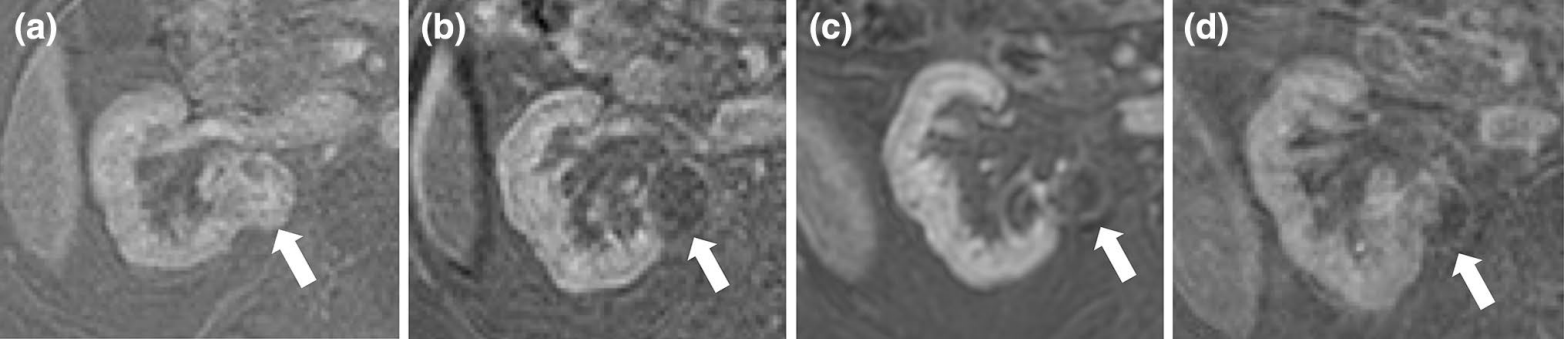
Tumor recurrence after cryoablation of a RCC. **a** Pre-ablation fat-suppressed T1-weighted CE MRI shows **a** 3.0 cm enhancing mass in the mid portion of the right kidney, posterior to the renal vein (arrow). **b**, **c** Follow-up CE MRI 6 months and 1 year after cryoablation show lack of enhancement within the ablation zone (arrows). **d** Follow-up CE MRI 2 years after cryoablation. In the ablated area (arrow) there is lack of enhancement medially and new enhancement in the lateral aspect consistent with tumor recurrence

**Fig. 5:**
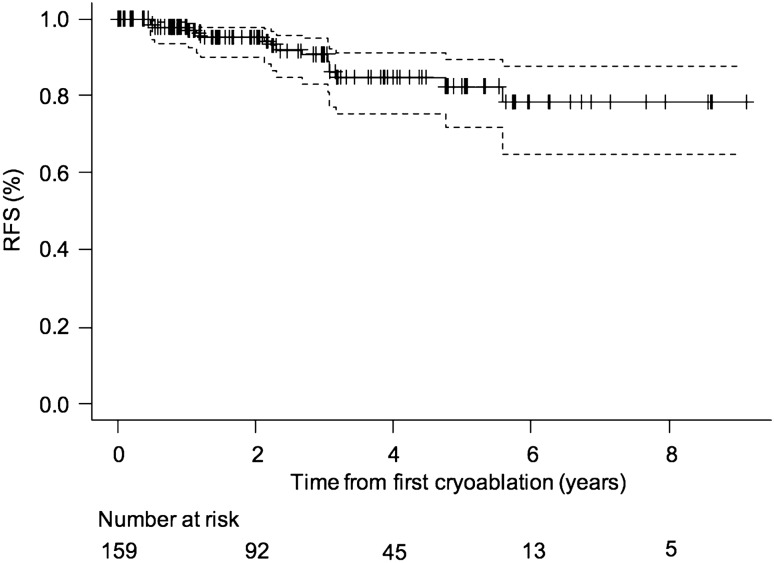
Kaplan–Meier curve of recurrence-free survival (RFS) in 159 patients with 159 biopsy-proven renal cell carcinomas. Dashed lines = 95% confidence intervals. Tick marks = censored data. Estimated RFS rates: 3 years: 90.5% (83.0%–94.9%). 5 years: 82.4% (72.0%–89.4%)

**Fig. 6:**
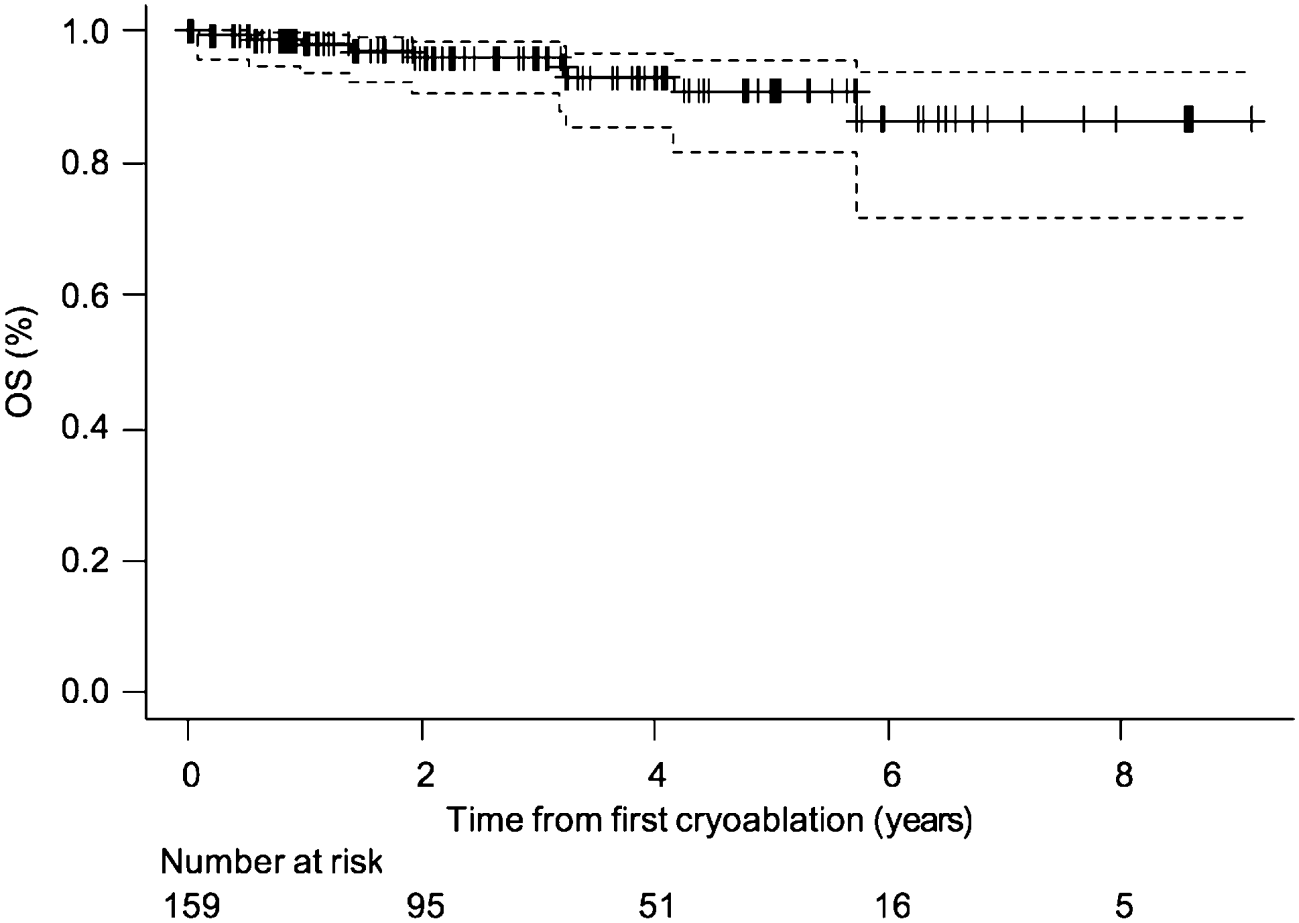
Kaplan–Meier curve of overall survival (OS) in 159 patients with 159 biopsy proven renal cell carcinomas. Dashed lines = 95% confidence intervals. Tick marks = censored data. Estimated OS rates: 3 years: 96.0% (90.6%–98.3%). 5 years: 91.0% (81.7%–95.7%)

The 159 patients with 159 biopsy proven RCCs had a median follow-up of 26.9 months (range 1-109 months) (Fig. 3). In this group of patients 16 recurrences (10%) were recorded at different time intervals after treatment, namely 6 months after treatment (3 patients), one year after (3 patients), two years after (3 patients) (Fig. 4), three years after (5 patients), five years after (2 patients). 10 recurrencies were successfully retreated with cryoablation, 2 of them were successfully surgically treated and 4 of them did not undergo any treatment after multidisciplinary evaluation. RFS rates were 90.5% (95% CI 83.0%, 94.9%) at 3 years and 82.4% (95% CI 72.0%, 89.4%) at 5 years (Fig. 5). RFS rates did not significantly differ between the 4 centers (p = 0.10, Log-Rank test). None of the variables we considered correlated with RFS (Table 4). OS rates were evaluated in the 159 patients with 159 biopsy proven RCCs. OS rates were 96.0% (95% CI 90.6%, 98.3%) at 3 years and 91.0% (95% CI 81.7%, 95.7%) at 5 years (Fig. 6).

**Table 4:** Univariate Cox regression analysis of recurrence-free survival in 159 patients with biopsy proven renal cell carcinoma

Variables	Hazard ratio (95% CI)	p-value
*Center*		
n. 1	1.00 (Reference)	
n. 2	1.59 (0.53–4.78)	0.41
n. 3	0.36 (0.04–3.11)	0.36
n. 4	0.67 (0.08–5.74)	0.71
*Gender*		
Male	1.00 (Reference)	
Female	0.63 (0.22–1.73)	0.37
Age	0.99 (0.94–1.05)	0.83
BMI	1.03 (0.88–1.20)	0.72
ASA SCORE	1.27 (0.59–2.74)	0.62
*ASA*		
ASA 1–2	1.00 (Reference)	
ASA 3–4	1.46 (0.53–4.02)	0.46
Tumor size	1.02 (0.97–1.07)	0.52
*Tumor size*		
<25 mm	1.00 (Reference)	
> = 25 mm	2.16 (0.75–6.22)	0.14
*Location*		
Anterior	1.00 (Reference)	
Posterior	1.80 (0.51–6.32)	0.36
*Location*		
Endophytic	1.00 (Reference)	
Esophytic	1.23 (0.26–5.73)	0.79
Partially esophytic	1.27 (0.24–6.56)	0.77
*PADUA SCORE*		
6–7	1.00 (Reference)	
8–9	0.61 (0.20–1.82)	0.38
> = 10	1.38 (0.29–6.49)	0.68
*Histotype*		
Papillary	1.00 (Reference)	
Clear cell	3.06 (0.70–13.58)	0.14
Cromophobe	0.008 (0.00–inf)	0.99
*Anesthesia*		
Local	1.00 (Reference)	
Sedation	0.35 (0.09–1.33)	0.12
General–Laparo	0.13 (0.01–1.44)	0.10
General–Percutaneous	0.0001 (0.00–NA)	0.99
*Approach*		
Laparoscopic	1.00 (Reference)	
Percutaneous	0.40 (0.05–3.03)	0.37
*Number of cryoprobes*	0.93 (0.60–1.44)	0.75
Baseline Serum Creatinine (mg/dl)	0.42(0.06–2.73)	0.36
*Baseline Serum Creatinine*		
<1.30 mg/dl	1.00 (Reference)	
> = 1.30 mg/dl	0.32 (0.04–2.45)	0.27

*BMI* Body Mass Index, *ASA* American Society of Anesthesiology, *CI* Confidence Interval

MFS rate was not evaluated because no patient in the subset with 159 biopsy proven RCCs developed metastatic disease.

**Fig. 7:**
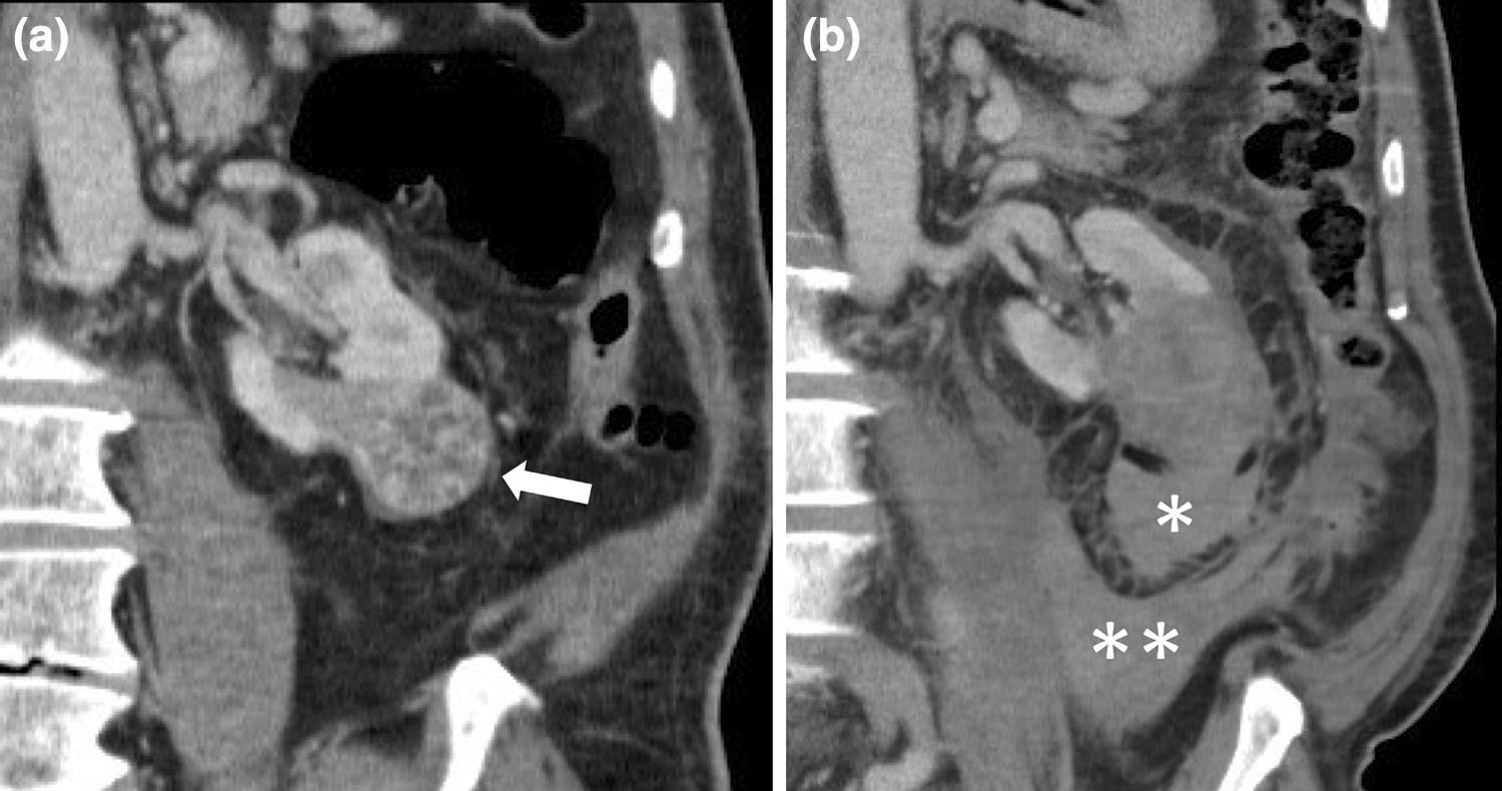
Hemorrhage after cryoablation of a RCC. **a** Pre-ablation CE CT shows a 3.5 cm enhancing mass at the lower pole of the left kidney (arrow). **b** CE CT obtained 1 day after cryoablation shows a perirenal hematoma (*) adjacent to the ablated area together with a pararenal hematoma (**). The patient recovered following transfusion

The overall complication rate was 18.4% (68/369 procedures, after considering 338 first procedures and 31 retreatments). 54/68 complications were graded as 1 according to the Clavien-Dindo classification (most of them minor, clinically insignificant, perirenal hematomas). Clavien-Dindo >1 complications were recorded in 14/369 procedures (3.8%) (Table 5), namely 11 grade 2 (Fig. 7) and 3 grade 3 complications (3.0% and 0.8%, respectively). No grade 4 and 5 complications were experienced. Recording of grade 1 complications appeared different between centers, as some centers recorded very minor hematomas, some of them not. Therefore statistical analysis considered Clavien-Dindo >1 complications only. Age >70 years (p = 0.04), tumor size >4 cm (p = 0.002) and use of >2 cryoprobes (p = 0.01) were related with Clavien-Dindo >1 complications.

**Table 5:** Clavien–Dindo greater than 1 complications of 369 renal cryoablations

Complication grade	N. and description
2	4 bleedings requiring transfusion 2 pneumothoraces requiring oxygen therapy 2 urinary tract infections requiring medical therapy 1 pleuric effusion requiring oxygen therapy 1 hypotension requiring medical therapy 1 hypertension requiring medical therapy
3a	1 pneumothorax requiring chest tube insertion
3b	1 ureteral stenting for clots in the excretory pathway 1 laparoscopy for peritoneal bleeding

Pre- and post-treatment (24-48 hours) serum creatinine was available in 328 patients and data analysis showed it significantly increased over baseline (pre-treatment median serum creatinine 1.02 mg/dl, range 0.50-7.00 mg/dl vs post-treatment median serum creatinine 1.07 mg/dl, range 0.54-8.91 mg/dl, p<0.001). Serum creatinine was unaffected by the procedure in 306/328 patients (93.3%), while in 22/328 patients (6.7%) serum creatinine increased >/ 0.3 mg/dl or >/ 1.5 times over baseline, suggesting an acute kidney injury [18]. No patient required dialysis after the treatment.

An additional analysis was restricted to the 340 percutaneously treated patients (without considering 23 laparoscopic cryoablations) and is available in the Online Resources. Results in terms of study’s outcomes (treatment efficacy, RFS and OS) were all confirmed. Two Clavien-Dindo 2 complications were recorded in the laparoscopically ablated patients group.

## Discussion

Treatment efficacy in our series of 338 patients with 363 renal tumors who underwent cryoablation was 95.9% after the first treatment and 98.1% after the second treatment. Such results are similar to those in recent studies [8, 10]. It is worth noting that such results were not collected in a single center with large specific experience but in four teaching and non-teaching general hospitals using the same technique. Therefore they underscore the intrinsic efficacy of the procedure.

Treatment efficacy results in the 159 patients with 159 biopsy proven RCCs who had a median follow-up of 26.9 months showed 16 recurrences (10%). Again these data are comparable to recent series. Cronan et al. [19] considered 17 articles including 2320 lesions and showed an overall recurrence rate of 8.1%. Our 159 patients with 159 biopsy proven RCCs had RFS rates of 90.5% (95% CI 83.0%, 94.9%) at 3 years and 82.4% (95% CI 72.0%, 89.4%) at 5 years. These rates are slightly lower compared to some recent series considering percutaneous cryoablation. Georgiades and Rodriguez [9] and Breen et al. [8] reported RFS rates of 97% and 93.9% at 5 years, respectively, Thompson et al. [20] reported an RFS rate of 98% at 3 years, while poorer results were reported by Kim et al. [21] and by Zargar et al. [22], that is 86.3% and 80% at 5 years, respectively. We might argue that our results are possibly related to the learning curve that all 4 centers experienced. However, OS rates in our patients were 96.0% (95% CI 90.6%, 98.3%) at 3 years and 91.0% (95% CI 81.7%, 95.7%) at 5 years and nobody developed metastatic disease. Such results are slightly better compared to other studies [8], and are possibly related to the different population (i.e. the mean diameter of the lesions was 2.6 cm in our series and 3.3 cm in the series by Breen et al. [8]). Of note, no patient experienced metastasis and the possibility of an immune stimulation of cryoablation on this regard should be kept in mind [23].

Statistical analysis showed a significant effect of ASA score on treatment efficacy, thus suggesting poorer efficacy results in more fragile patients. Treatment efficacy was not significantly affected by tumor location (anterior vs posterior, esophytic vs endophytic), in agreement with previous studies [8]. However, there could be a selection bias on this regard, due to more patients with favorably posteriorly located tumors being selected for this procedure. Additionally, PADUA score, which collects a number of location features, significantly affected treatment efficacy results. Tumor size and the number of cryoprobes (which is tumor size related) significantly affected treatment efficacy and such relationships appear reasonable, although tumor size was unrelated to treatment efficacy in the series by Breen et al. [8]. However, tumor size did not correlate with RFS rates, in agreement with other series [8, 10]. Additionally, RFS rates were not significantly affected by tumor histotype, while Beksac et al. [24] found an association between clear cell histology and progression. It is worth noting that treatment efficacy was not significantly affected by the type of anesthesia, including local anesthesia only.

The rate of recorded major complications in this series was extremely low, that is 0.8% (3/394 procedures) when considering Clavien-Dindo 3 or more, and compares favorably with data from the Literature. Major complications rate (Clavien-Dindo 3 or more) of 3.4% over 713 procedures were recently reported by Garnon et al. [25] considering a multicenter experience and higher rates were reported by Breen et al. [8], by Aoun et al. [10] and by Schmit et al. [26], that is 4.9% over 473 procedures, 2.8% over 357 procedures and 7.5% over 398 procedures, respectively. Georgiades [27] reported major complication rates between 3%-7% in different series. We recorded one single pneumothorax requiring chest tube insertion and two post-procedural bleedings requiring intervention. Extensive use of oblique imaging for small size (17G) cryoprobes guidance together with large use of hydrodissection for protection of adjacent vulnerable structures may potentially explain these results. Therefore, while minor bleeding is inevitable in the majority of procedures on the kidney [14], it appears that fear of significant hemorrhagic complications of cryoablation is probably not justified [28].

Age, tumor size and number of cryoprobes correlated with Clavien-Dindo >1 complications in our series. As regards the relationship between tumor size and complications, Aoun et al. [10] showed that tumors >3 cm were associated with greater incidence of major complications, while Garnon et al. [25] showed a significant higher risk of major complications for tumors >4 cm. However, Breen et al. [8] showed that tumors >4 cm did not prove significant in anticipating major complications. In our series, the mean diameter of the 363 treated lesions was 2.5 cm, thus smaller compared to other series (2.9 cm in the series by Aoun et al. [10], 2.8 cm in the series by Garnon et al. [25], 3.3 cm in the series by Breen et al. [8]). This feature could partly explain our low complication rate.

It is worth noting that a minority of our percutaneous cryoablations were performed under general anesthesia (30/340, 8.8%) while 234/340 cryoablations (68.8%) were performed after conscious sedation and 76 (22.3%) under local lidocaine anesthesia only. As we did not anticipate local anesthesia to play a significant role in the insurgence of complications, we did not systematically use general anesthesia, contrary to the Guidelines of CIRSE [14]. However, these Guidelines consider all types of percutaneous ablation of renal carcinomas, including radiofrequency and microwave, that are definitely more painful. While admittedly, general anesthesia reduces intraoperative patient awareness and recall and offers pain control for prolonged periods of time, thereby allowing for more complex interventions [14], our approach was less invasive, allowing a more sparing use of material and personnel. A number of studies showed that cryoablation under local anesthesia and conscious sedation was as safe and effective as cryoablation under general anesthesia [11–13]. In center 1 anesthesiologists considered unfit for treatment with local anesthesia only uncooperative patients or patients who were possibly unable to keep prone position for the entire length of cryoablation. Therefore they did not take part in the last 71 percutaneous procedures performed under local anesthesia only. In this setting, patient awareness allowed immediate perception of one malfunctioning cryoprobe. However, in 2 cases the patient treated with local anesthesia only could no longer tolerate the treatment after a long procedure with treatment of multiple tumors. Therefore, conscious sedation might be preferable to local anesthesia when multiple tumor treatments at the same time are planned. Furthermore, we agree that the percutaneous approach should be preferred when feasible because it reduces pain, hospitalization length and costs [29].

Cryoablation was not associated with a significant decrease in renal function after treatment in 93.3% of the patients in our series, in line with the literature. The safety of cryoablation with regard to renal function has been well documented in multiple studies [27]. Zondervan et al. [30] stated in their review that cryoablation entails minimal deterioration of the renal function, although chronic kidney disease stage progression may occur in 25% of patients. Glomerular filtration rate did not significantly change in the series by Aoun et al. [10] and such result is possibly attributable to excellent visualization of approximately 1 cm of ice beyond the tumor margin.

High-quality evidence comparing different thermal ablation techniques is missing and Uhlig et al. [1] stated that comparisons between radiofrequency ablation, cryoablation and microwave ablation have not been established so far. Precise visualization of the ice ball under CT guidance potentially allows safer cryoablation treatment of critically located lesions (i.e. close to the ureter or to the bowel). Additionally, cryoablation offers the possibility of treatment under local anesthesia only, contrary to the other techniques. On the other hand, cryoablation is longer and entails higher costs.

A number of studies comparing cryoablation with partial nephrectomy contributed to meta-analyses. A recent network meta-analysis [1] showed all-case mortality was higher for cryoablation (but patients were older and had more comorbidities), cancer-specific mortality did not show significant differences, local recurrence was higher for cryoablation and the likelihood of complications was lower for cryoablation while there was no significant difference in renal function changes. A lack of high-quality evidence does not allow definite conclusions regarding morbidity and oncologic outcomes of cryoablation [3]. On the other hand, evidence-based medicine considers patient preferences as well [31]. Candidates to cryoablation should be informed about the higher risk of local recurrence compared to partial nephrectomy [3] and the possibility of recurrence retreatment with cryoablation. Additionally, future surgery is not precluded.

There are limitations to the present study. Data were retrospectively collected. Some laparoscopic procedures were considered although their number was too small for statistical assessment. The same applies to the small number of stage T1b tumors. Radiology reports of follow-up exams served as reference standard and follow-up images were not reviewed by independent radiologists for consensus read. A standard of reference for confirming no recurrence evidenced by absence of a new enhancement of the ablated lesion during follow-up is lacking. Additionally, some patients were lost to follow-up and patient renal function outcome was not recorded.

In conclusion, despite these limitations, cryoablation performed across four different centers in a large cohort of predominantly small renal tumors showed this technique provides good recurrence-free survival rates and overall survival rates at three- and five-year with very low major complications rate. These results are comparable with large single-institutional data and were obtained without use of general anesthesia.

## Supplementary Information

Below is the link to the electronic supplementary material.

Supplementary material 1 (DOCX 15 kb)

Supplementary material 2 (DOCX 141 kb)

Supplementary material 3 (DOCX 20 kb)

Supplementary material 4 (DOCX 173 kb)

Supplementary material 5 (PDF 107 kb)

Supplementary material 6 (PDF 106 kb)

## Data Availability

The authors declare that the data supporting the findings of this study are available within the article and its supplementary information files.
