# Supplementary material for: Cryoablation of renal tumors: long-term follow-up from a multicenter experience
Source: Abdom Radiol (NY). 2021 Apr 29;46(9):4476–88. doi: 10.1007/s00261-021-03082-z (PMC8346457; doi:10.1007/s00261-021-03082-z)
Supplement: Supplementary file 1 — Supplementary material 1 (DOCX 15 kb) [file 261_2021_3082_MOESM1_ESM.docx]

**Article title:** cryoablation of renal tumours: long-term follow-up from a multicentre experience
**Journal name:** Abdominal Radiology
**Author names:** Fulvio Stacul, Camilla Sachs, Fabiola Giudici, Michele Bertolotto, Michele Rizzo, Nicola Pavan, Luca Balestreri, Oliviero Lenardon, Alessandro Pinzani, Lisa Pola, Calogero Cicero, Antonio Celia, Maria Assunta Cova

**Affiliation and e-mail address of corresponding author:** Maria Assunta Cova, Department of Radiology, University of Trieste, Trieste, Italy**.** E-mail: m.cova@fmc.units.it.

**Tab 1:** Clinical features for percutaneously treated patients

|  | **All patients**  **(n=315)** | **Patients with biopsy proven RCC**  **(n=142)** |
| --- | --- | --- |
| **Age**  Mean (SD)  Median (Min-Max) | 73 (9)  75 (39-90) | 75 (9)  76 (43-90) |
| **Gender**  Male (n, %)  Female (n, %) | 227 (72.1%)  88 (27.9%) | 102 (71.8%)  40 (28.2%) |
| **BMI**  Mean (SD)  Median (Min-Max) | 26.5 (3.7) 26.2 (18.6-40.1) | 25.8 (3.1)  25.6 (18.6-36) |
| **ASA**  Mean (SD)  Median (Min-Max) | 2.6 (0.6)  3 (1-4) | 2.5 (0.6)  3 (1-4) |
| **ASA**  ASA score 1 (n, %)  ASA score 2 (n, %)  ASA score 3 (n, %)  ASA score 4 (n, %) | 13 (4.1%)  119 (37.8%)  170(54.0%)  13 (4.1%) | 8 (5.6%)  54 (38.0%)  77 (54.2%)  3 (2.1%) |
| Single kidney (n, %)  Transplanted Kidney (n, %) | 36 (11.4%)  1 (0.32%) | 0 (0.0%)  1 (0.70%) |
| History of RCC  No (n, %)  Yes (n, %)  Hereditary syndrome (n, %) | 275 (87.3%)  29 (9.2%)  11 (3.5%) | // |

*RCC:* Renal Cell Carcinoma; *SD*: Standard Deviation; *BMI*: Body Mass Index; *ASA*; American Society of Anesthesiology.
