# Supplementary material for: Cryoablation of renal tumors: long-term follow-up from a multicenter experience
Source: Abdom Radiol (NY). 2021 Apr 29;46(9):4476–88. doi: 10.1007/s00261-021-03082-z (PMC8346457; doi:10.1007/s00261-021-03082-z)
Supplement: Supplementary file 2 — Supplementary material 2 (DOCX 141 kb) [file 261_2021_3082_MOESM2_ESM.docx]

**Article title:** cryoablation of renal tumours: long-term follow-up from a multicentre experience
**Journal name:** Abdominal Radiology
**Author names:** Fulvio Stacul, Camilla Sachs, Fabiola Giudici, Michele Bertolotto, Michele Rizzo, Nicola Pavan, Luca Balestreri, Oliviero Lenardon, Alessandro Pinzani, Lisa Pola, Calogero Cicero, Antonio Celia, Maria Assunta Cova

**Affiliation and e-mail address of corresponding author:** Maria Assunta Cova, Department of Radiology, University of Trieste, Trieste, Italy**.** E-mail: m.cova@fmc.units.it.

**Tab.2** Tumour characteristics for 315 percutaneously treated patients with renal masses and for the subset of 142 patients with biopsy proven renal cell carcinoma

|  | **All patients**  **(n=315)** | **Patients with biopsy proven RCC**  **(n=142)** |
| --- | --- | --- |
| **Tumour size (mm)**  Mean (SD)  Median (Min-Max) | 25.6 (9.6)  25 (6-53) | 26.8 (9.7)  25 (10-53) |
| **Location**  Anterior (n, %)  Posterior (n, %) | 76 (24.1%)  239 (75.9%) | 37 (26.1%)  105 (73.9%) |
| **Location**  Endophytic (n, %)  Esophytic (n, %)  Partially esophytic (n, %) | 62 (19.7%)  144 (45.7%)  109 (34.6%) | 32 (22.5%)  60 (42.3%)  50 (32.2%) |
| **Padua Score**  Mean (SD)  Median (Min-Max) | 7.9 (1.4)  8 (6-12) | 8.0 (1.4)  8 (6-12) |
| **Padua Score**  6-7 (n, %)  8-9 (n, %)  >=10 (n, %) | 140(44.7%)  130 (41.5%)  43 (13.7%) | 55 (39.0%)  67 (47.5%)  19 (13.5%) |
| **N° of tumours**  **1** (n, %)  **>1** (n, %) | 306 (97.1%)  9 (2.9%) | 142 (100.0%)  0 (0.0%) |
| **Histotype**  Clear Cells (n, %)  Papillary (n, %) Chromophobe (n, %)  Unspecified adenocarcinoma (n, %) | // | 93 (66.2%)  30 (21.1%)  9 (6.3%)  9 (6.3%) |

*RCC:* Renal Cell Carcinoma; *SD*: Standard Deviation
