# Supplementary material for: Cryoablation of renal tumors: long-term follow-up from a multicenter experience
Source: Abdom Radiol (NY). 2021 Apr 29;46(9):4476–88. doi: 10.1007/s00261-021-03082-z (PMC8346457; doi:10.1007/s00261-021-03082-z)
Supplement: Supplementary file 3 — Supplementary material 3 (DOCX 20 kb) [file 261_2021_3082_MOESM3_ESM.docx]

**Article title:** cryoablation of renal tumours: long-term follow-up from a multicentre experience
**Journal name:** Abdominal Radiology
**Author names:** Fulvio Stacul, Camilla Sachs, Fabiola Giudici, Michele Bertolotto, Michele Rizzo, Nicola Pavan, Luca Balestreri, Oliviero Lenardon, Alessandro Pinzani, Lisa Pola, Calogero Cicero, Antonio Celia, Maria Assunta Cova

**Affiliation and e-mail address of corresponding author:** Maria Assunta Cova, Department of Radiology, University of Trieste, Trieste, Italy**.** E-mail: m.cova@fmc.units.it.

**Tab.3** Analysis of variables predicting treatment efficacy of 340 percutaneously treated renal tumours

| **Variables** | **Treatment Efficacy after Primary Ablation**  **(n=326)** | **No Treatment Efficacy after Primary Ablation**  **(n=14)** | **p-value** |
| --- | --- | --- | --- |
| **Center**  **n. 1 (n, %)**  **n. 2 (n, %)**  **n. 3 (n, %)**  **n. 4 (n, %)** | 135 (95.1%)  44 (100.0%)  78 (94.0%)  69 (97.2%) | 7 (4.9%)  0 (0.0%)  5 (6.0%)  2 (2.8%) | 0.36 |
| **Gender**  Male **(n, %)**  Female **(n, %)** | 237 (96.7%)  89 (93.7%) | 8 (3.3%)  6 (6.3%) | 0.20 |
| **Age**  Median (Min-Max) | 74.9 (39.1-89.4) | 73.3 (45.6-89.6) | 0.89 |
| **BMI**  Median (Min-Max) | 26.2 (18.6-40.1) | 27.0 (20.5-37.4) | 0.89 |
| **ASA SCORE**  <4 **(n, %)**  >=4 **(n, %)** | 314 (96.3%)  12 (85.7%) | 12 (3.7%)  2 (14.3%) | 0.05 |
| **Single kidney or transplanted kidney**  Yes **(n, %)**  No **(n, %)** | 43 (89.6 %)  283 (96.9%) | 5 (10.4%)  9 (3.1%) | 0.01* |
| **History of RCC**  No **(n, %)**  Yes **(n, %)**  Hereditary Syndrome **(n, %)** | 257 (96.3 %)  39 (95.1%)  30 (94.0 %) | 10 (3.7%)  2 (4.9%)  2 (6.0%) | 0.58 |
| **Tumour size (mm)**  **Median (Min-Max)**  <25mm **(n, %)**  >=25mm **(n, %)** | 25 (6-53)  161 (98.8%)  165 (93.2%) | 35 (15-50)  2 (1.2%)  12 (6.8%) | 0.01* |
| **Location**  Endophytic **(n, %)**  Esophytic **(n, %)**  Partially esophytic **(n, %)** | 72 (96.0%)  147 (98.0 %)  107 (93.0%) | 3 (4.0%)  3 (2.0%)  8 (7.0%) | 0.13 |
| **PADUA SCORE**  **Median (Min-Max)**  <9 **(n, %)**  >=9 **(n, %)** | 8 (6-12)  225 (97.0%)  99 (92.5%) | 9 (7-11)  6 (3.0%)  8 (7.5%) | 0.04* |
| **Number of tumours for treatment session**  1 **(n, %)**  >1 **(n, %)** | 305 (96.2%)  21 (91.3%) | 12 (3.8%)  2 (8.7%) | 0.25 |
| **Anesthesia**  Local **(n, %)**  Sedation **(n, %)**  General **(n, %)** | 74 (97.4%)  223 (95.3%)  29 (96.7%) | 2 (2.6%)  11 (4.7%)  1 (3.3%) | 0.71 |
| **Number of cryoprobes**  **Median (Min-Max)**  <=2 **(n, %)**  >2 **(n, %)** | 2 (1-8)  199 (97.6%)  127 (93.4%) | 3 (2-7)  5 (2.4%)  9 (6.6 %) | 0.05* |
| **Baseline Serum Creatinine (mg/dl)**  Median (Min-Max) | 1.02 (0.50-6.2) | 1.10 (0.51-7.0) | 0.08 |
| **Baseline Serum Creatinine****  <1.30 mg/dl **(n, %)**  >=1.30 mg/dl **(n, %)** | 245 (96.8%)  67 (93.1%) | 8 (3.2%)  5 (6.9%) | 0.15 |

*** 15 missing data*

*RCC:* Renal Cell Carcinoma; *BMI*: Body Mass Index; *ASA*; American Society of Anesthesiology.
