# Supplementary material for: Cryoablation of renal tumors: long-term follow-up from a multicenter experience
Source: Abdom Radiol (NY). 2021 Apr 29;46(9):4476–88. doi: 10.1007/s00261-021-03082-z (PMC8346457; doi:10.1007/s00261-021-03082-z)
Supplement: Supplementary file 4 — Supplementary material 4 (DOCX 173 kb) [file 261_2021_3082_MOESM4_ESM.docx]

**Article title:** cryoablation of renal tumours: long-term follow-up from a multicentre experience
**Journal name:** Abdominal Radiology
**Author names:** Fulvio Stacul, Camilla Sachs, Fabiola Giudici, Michele Bertolotto, Michele Rizzo, Nicola Pavan, Luca Balestreri, Oliviero Lenardon, Alessandro Pinzani, Lisa Pola, Calogero Cicero, Antonio Celia, Maria Assunta Cova

**Affiliation and e-mail address of corresponding author:** Maria Assunta Cova, Department of Radiology, University of Trieste, Trieste, Italy**.** E-mail: m.cova@fmc.units.it.

**Tab. 4** Univariate Cox regression analysis of recurrence-free survival in 142 percutaneously treated patients with biopsy proven renal cell carcinoma

| **Variables** | **Hazard Ratio (95% CI)** | **p-value** |
| --- | --- | --- |
| **Center**  **n. 1**  **n. 2**  **n. 3**  **n. 4** | 1.00 (Reference)  0.36 (0.04-3.13) 0.66 (0.08-5.66)  2.26 (0.74-6.96) | 0.36  0.70  0.15 |
| **Gender**  Male  Female | 1.00 (Reference)  0.62 (0.22-1.74) | 0.36 |
| **Age** | 0.99 (0.93-1.05) | 0.78 |
| **BMI** | 1.04 (0.89-1.21) | 0.64 |
| **ASA SCORE** | 1.35 (0.60-3.05) | 0.46 |
| **ASA**  **ASA 1-2**  **ASA 3-4** | 1.00 (Reference)  1.47 (0.52-4.17) | 0.46 |
| **Tumour size** | 1.01 (0.96-1.06) | 0.75 |
| **Tumour size**  **<25mm**  **>=25mm** | 1.00 (Reference)  1.87 (0.63-5.94) | 0.26 |
| **Location**  Anterior  Posterior | 1.00 (Reference)  2.05 (0.46-9.12) | 0.35 |
| **Location**  Endophytic  Esophytic  Partially esophytic | 1.00 (Reference)  1.58 (0.34-7.34)  1.11 (0.20-6.06) | 0.56  0.91 |
| **PADUA SCORE**  **6-7**  **8-9**  **>=10** | 1.00 (Reference)  0.41 (0.13-1.45)  1.08 (0.23-5.09) | 0.14  0.92 |
| **Histotype**  Adenocarcinoma  Papillary  Clear Cell  Cromophobe | 1.00 (Reference)  0.67 (0.06-7.37)  1.61 (0.21-12.45)  0.008 (0.00-inf) | 0.74  0.65  0.99 |
| **Anesthesia**  Local  Sedation  General | 1.00 (Reference)  0.35 (0.09-1.36)  0.0004 (0.00-NA) | 0.13  0.99 |
| **Number of cryoprobes** | 0.92 (0.59-1.44) | 0.71 |
| **Baseline Serum Creatinine (mg/dl)** | 0.42(0.06-2.67) | 0.35 |
| **Baseline Serum Creatinine**  <1.30 mg/dl  >=1.30 mg/dl | 1.00 (Reference)  0.31 (0.04-2.37) | 0.26 |

*BMI*: Body Mass Index; *ASA*; American Society of Anesthesiology; *CI*: Confidence Interval.
