# Supplementary material for: Cryoablation of renal tumors: long-term follow-up from a multicenter experience
Source: Abdom Radiol (NY). 2021 Apr 29;46(9):4476–88. doi: 10.1007/s00261-021-03082-z (PMC8346457; doi:10.1007/s00261-021-03082-z)

Article title: cryoablation of renal tumours: long-term follow-up from a multicentre experience

Journal name: Abdominal Radiology

Author names: Fulvio Stacul, Camilla Sachs, Fabiola Giudici, Michele Bertolotto, Michele Rizzo, Nicola Pavan, Luca Balestreri, Oliviero Lenardon, Alessandro Pinzani, Lisa Pola, Calogero Cicero, Antonio Celia, Maria Assunta Cova

Affiliation and e-mail address of corresponding author: Maria Assunta Cova, Department of Radiology, University of Trieste, Trieste, Italy. E-mail: m.cova@fmc.units.it.

Fig. 1 Kaplan-Meier curve of recurrence-free survival (RFS) in 142 percutaneously treated patients with 142 biopsy-proven renal cell carcinomas. Dashed lines = 95% confidence intervals. Tick marks = censored data.

Estimated RFS rates:

3 years: 90.5% (82.2%-94.9%)

5 years: 81.0% (68.7%-88.8%)

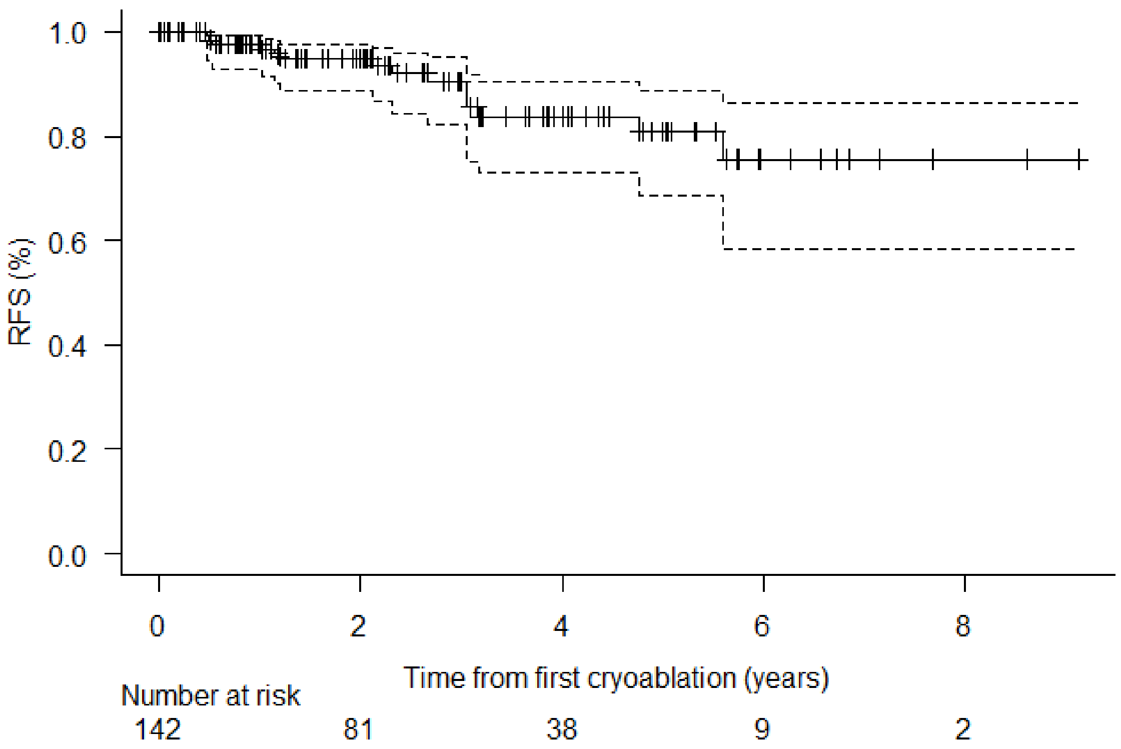

Supplement: Supplementary file 5 — Supplementary material 5 (PDF 107 kb) [file 261_2021_3082_MOESM5_ESM.pdf]
